# The Nature of Cough

**Published:** 1856-08

**Authors:** 


### THE NATURE OF COUGH,

Is an instinctive spasmodic effort of the lungs to expel the air which they contain, through their "pipes," or their bronchial branches, for the purpose of carrying before it, and out through the mouth, any thing which is in the lungs or air passages, and which ought not to be there. It is a law of the animal economy to relieve itself; not the least of all the wonderful adaptations which infinite wisdom and benevolence has ordained for our preservation. The eye begins to water and wash out with tears the particle of dust or sand which offends it. The stomach revolts instantaneously at the presence of poison, and rejects it. The tongue repels anything placed upon it, that is not adapted to the well being of the system. And if the lungs were less vigilant, accumulations would take place from time to time, and they would eventually fill with solid substances, air could not enter, and we would die. Cough is excited by putting a straw or feather or other offending substance in the ear; thus if a person is asleep, and an insect were crawling in, the cough would arouse him.

Cough is the common attendant of consumptive disease. Although it does not imply that because a man has a cough he must necessarily have consumption, yet no one can have consumption without a cough, sooner or later, with extremely rare exceptions.

This cough is an effort of nature to remove from the lungs that which ought not to be there, that which is causing mischief, just as vomiting is an effort of nature to remove from the stomach that which, if permitted to remain longer, would cause increasing mischief. Therefore to take medicine to repress cough is to counteract nature, and if persevered in, will always hasten death. Hence opium, paregoric, laudanum, morphine, or any other anodyne known to men, when taken day after day, will inevitably and under all circumstances make death the more certain in all forms of consumptive disease, unless there is a physician in attendance to counteract their mischievous effects. And as every intelligent druggist knows that of all the patent or secret medicines sold for coughs, colds and consumption, there is not a single one that does not contain an opiate or anodyne in some shape or form, so they all fight against nature, derange her machinery, lock up the glands of the system, disorder the secretories, and therefore must prepare the way for a more certain decline and death. It is therefore suicidal to use them.

